# RILES, a novel method for temporal analysis of the *in vivo* regulation of miRNA expression

**DOI:** 10.1093/nar/gkt797

**Published:** 2013-09-05

**Authors:** Safia Ezzine, Georges Vassaux, Bruno Pitard, Benoit Barteau, Jean-Marc Malinge, Patrick Midoux, Chantal Pichon, Patrick Baril

**Affiliations:** ^1^Centre de Biophysique Moléculaire, CNRS UPR4301, Université d’Orléans and Inserm, Orléans, France, ^2^UMRE 4320, Faculté de Médecine, Université de Nice-Sophia-Antipolis, Nice, France, ^3^Inserm UMR 1087/CNRS UMR 6291, Université de Nantes, Faculté de médecine, L’institut du Thorax, Nantes F-44000 and ^4^In-Cell-Art, Nantes F44200, France

## Abstract

Novel methods are required to investigate the complexity of microRNA (miRNA) biology and particularly their dynamic regulation under physiopathological conditions. Herein, a novel plasmid-based RNAi-Inducible Luciferase Expression System (RILES) was engineered to monitor the activity of endogenous RNAi machinery. When RILES is transfected in a target cell, the miRNA of interest suppresses the expression of a transcriptional repressor and consequently switch-ON the expression of the luciferase reporter gene. Hence, miRNA expression in cells is signed by the emission of bioluminescence signals that can be monitored using standard bioluminescence equipment. We validated this approach by monitoring in mice the expression of myomiRs-133, −206 and −1 in skeletal muscles and miRNA-122 in liver. Bioluminescence experiments demonstrated robust qualitative and quantitative data that correlate with the miRNA expression pattern detected by quantitative RT-PCR (qPCR). We further demonstrated that the regulation of miRNA-206 expression during the development of muscular atrophy is individual-dependent, time-regulated and more complex than the information generated by qPCR. As RILES is simple and versatile, we believe that this methodology will contribute to a better understanding of miRNA biology and could serve as a rationale for the development of a novel generation of regulatable gene expression systems with potential therapeutic applications.

## INTRODUCTION

MicroRNAs (miRNAs) are a class of endogenous noncoding RNAs, 18–25 nt in length, that posttranscriptionally regulate the expression of eukaryotic genes in a sequence-specific manner. miRNAs act by binding to mRNA targets, preferentially to the 3′-untranslated region (3′UTR) by a base-pairing mechanism. Depending on the degree of complementarity, miRNAs either inhibit translation or induce degradation of the target mRNA (1). To date, >1000 miRNAs have been identified in the human genome and they are predicted to regulate 60% of the whole transcriptome (2). MiRNAs are implicated in most, if not all, cellular processes from proliferation, apoptosis and differentiation, to hematopoiesis, developmental timing and organogenesis (1). Therefore, it is not surprising that deregulation of miRNAs has also been associated with a number of diseases and that RNAi-based therapeutic agents show promise as therapeutic drugs (3).

Current methods used to determine the expression of miRNAs have strongly impacted our knowledge of the biological roles that miRNAs play under physiological and pathophysiological conditions. While the data generated cannot be disputed, they lack spatial and, more importantly, temporal resolution. Methods such as PCR(-based approaches), microarrays, northern blot and ELISA are fully invasive and require complex tissue sampling and processing (4,5), making these procedures unsuitable for monitoring miRNA regulation during longitudinal studies. This is particularly problematic as miRNAs are spatiotemporally regulated and subject to considerable interindividual variation (6,7). This source of complexity is even more pronounced when the expression of miRNAs needs to be investigated at the whole-organism level. For instance, it is well established that miRNAs are finely regulated during embryonic development and control complex regulatory networks of gene expression involved in cell-lineage decisions and subsequently morphogenesis (8–10). Similarly, in cancer, some miRNAs are implicated in the early phases of tumor development while they can, at later stages, inhibit the formation of metastases (11,12). Therefore, the average measurement of miRNAs from a heterogeneous population at a specific time point underestimates the biological relevance of the time-dependent nature of miRNA regulation as well as the heterogeneity of miRNA expression at the individual level. Consequently, these data could result in the loss of important information connecting miRNA expression and cell function. Addressing these limitations can impact directly on basic and therapeutic research fields. Noninvasive molecular imaging methods have the potential to overcome these limitations (13) and to provide an alternative method to study miRNA expression under physiological conditions (14). However, the monitoring of miRNAs in real time, in a complex organism, is challenging primarily owing to the short length of miRNAs. This could explain the limited number of reports in the literature. The first reported method (15,16) is based on the use of the luciferase reporter gene carrying complementary block sequences to a specific miRNA in the 3′UTR of the luciferase gene. Therefore, when a miRNA of interest is expressed in the cell, it binds to the luciferase transcript and inhibits the production of luciferase. In this way, miRNA expression in cells is signed by a decrease in the bioluminescence signal (Off-System). However, such a ‘negative’ imaging modality is not adequate, as the loss of the bioluminescence signal may reflect nonspecific regulations of the luciferase promoter or even cell death. More recently, positive molecular imaging systems (ON-systems) have been developed to overcome this limitation. Some of these systems are based on the use of oligonucleotide molecular beacons labelled both with a fluorophore at one end and a quencher at the other end (16–22). In presence of a specific miRNA, the stem-loop structure of the beacons is linearized, separating the fluorophore from the quencher. As a result, the fluorescence signal emitted in cells was found to be proportional to the concentration of miRNAs. While this advance represents a more rational and *in vitro* fully validated approach (18), this methodology does have limitations, principally the weak sensitivity of fluorogenic probes in small animals and the restricted application to miRNA analysis expressed from cells implanted *in vivo* (20,21,23). Moreover, major complex normalization procedures are required because of the necessity of repeating administration of the probes in the course of longitudinal studies.

We developed a RNAi-Inducible Luciferase Expression System (RILES) with the aim of generating positive bioluminescence signals in mice that will allow the qualitative and quantitative measurement of endogenous expressed miRNAs with sufficient sensitivity to monitor the dynamic regulation of miRNA expression during the development of a chronic disease. For this purpose, we customized the recently characterized Cumate gene-switch inducible expression system (24). This system, like other repressor-based inducible expression systems (25,26), uses an inducer that, when bound to a repressor protein, changes its conformation, impeding the binding to an operator sequence located downstream to the translation start codon within a constitutive promoter. Consequently, the expression of the transgene is switched-ON by the presence of the exogenous inducer. We reasoned that placing expression of the repressor molecule directly under the control of the endogenous RNAi machinery, rather than an exogenous molecule, would be an alternative way to switch-ON expression of the transgene. Consequently, if the luciferase reporter gene is used as a transgene, the system will generate bioluminescence signals that will qualitatively and quantitatively reflect the expression pattern of miRNAs.

Here we report a complete proof of principle study and demonstrate that RILES provides specific and relevant biological information about the expression pattern and the temporal regulation of endogenous miRNA under physiological and pathological conditions.

## MATERIALS AND METHODS

### Plasmid construction

For the construction of RILES plasmids, we first restriction-digested and subcloned the firefly luciferase cDNA into the pCMV5/Cuo plasmid (Cumate™ inducible expression plasmid, Qbiogene, CA, USA). CymR cDNA from the pSV40/CymR plasmid (Qbiogene) was then PCR amplified, subcloned into pcDNA3/Topo TA (Invitrogen) and its functionality was tested on transfection of HEK 293 cells cultured in the presence of cumate as a gene-switch agent (24) (data not shown). The puromycin cDNA fragment driven by the SV40 promoter was removed from the pCMV5/Cuo/Fluc plasmid (Qbiogene) and replaced by CymR cDNA excised from pCMV/Topo/CymR. A multiple cloning site containing Mlu I and Not I restriction sites was inserted into the 3′UTR of CymR cDNA before the polyadenylation site. The sequence and the functionality of the expression plasmid generated were verified and tested functionally (24) (data not shown). To place the expression plasmid under control of a specific miRNA or a siRNA, we designed a 120-bp double-stranded oligonucleotide containing four blocks of complementary sequences to the miRNA or the siRNA. The oligonucleotides were flanked at the 3′-end by a compatible, overhanging, phosphorylated Not I sequence, and at the 5′-end by a compatible, overhanging, phosphorylated Nhe I sequence. The upper single-stranded oligonucleotide and the lower single-stranded oligonucleotide were synthesized separately (Eurogentec, Seraing, Belgium), annealed at 94°C for 5 min and then at 37°C for 60 min. The annealed oligonucleotides were ligated to the purified expression plasmid previously double-digested with Not I and Mlu I. All the vectors generated (Supplementary Table S1) were sequenced and amplified using Endofree plasmid kits (Qiagen).

### Cell lines, reagents and transfection

HEK 293, C2C12 and HuH7 cell lines were obtained from ATCC. The HLE cell line was kindly provided by Pascal Pineau (Institut Pasteur, Paris, France). Cells were cultured in 4 g/l Dulbecco's modified Eagle's medium supplemented with 10% SVF, and with penicillin and streptomycin. C2C12 cells were differentiated into myoblasts by culturing subconfluent cell monolayers in 2% horse serum for 4 days. Synthetic precursor miRNAs were obtained from Life technology, pU6/shRNA turbo GFP and the pU6/shRNA control plasmids were obtained from Sigma and the pQE30 expression plasmid was from Qiagen. Icafectin 441 (Eurogentec) and His-lPEI (27) were used as *in vitro* transfection reagents. For transfection, 1 × 10^5^ cells/well, in 24-well plates, were transfected with 2 µg/well of plasmid DNA and synthetic precursor miRNA (Invitrogen). The pQE30 empty expression plasmid was used to normalize transfection conditions. Relative luciferase units (RLU) were determined 48 (HEK 293 cells) and 72 h (HuH7, HLE, differentiated C2C12) after transfection using a luminometer (Berthold). Luciferase activities were normalized to protein content (RLU/mg protein) and expressed as fold induction relative to control cells transfected with the expression plasmid alone set to the arbitral value of 1.

### Immunohistochemistry

Immunodetection of luciferase protein in muscle tissues was performed using a specific luciferase antibody (Promega). A solution composed of 10 mM Tris/EDTA, pH 9, was used to unmask antigen sites from paraffin-embedded tissues.

### Quantitative reverse transcriptase-polymerase chain reaction and genomic polymerase chain reaction

Extraction of total RNA was performed by adding 1/20 (w/vol) volume of lysis binding buffer (mirVana microRNA isolation kit, Ambion) followed by tissue homogenization and gridding using CKMix ceramic-bead tubes (Ozyme, Paris, France) and the Precelyss 24 Unit (Precelyss, Bertin, France). RNA integrity was determined by calculating the RNA integrity number using a BioAnalyzer 2100 (Agilent technologies). Samples with an RNA integrity number superior or equal to 8 were considered for further analysis. For miRNA analysis, cDNA was synthesized using the NCode VILO miRNA cDNA synthesis kit according to the manufacturer's instructions (Invitrogen). This step adds a polyadenylate tail to the miRNA population within the total RNA samples. For mRNA analysis, 100 ng of total RNA samples was also used but reverse transcripted using the SuperScript II Reverse Transcriptase Kit (Invitrogen) as previously described (28). The real time quantitative PCR products were generated from 50 ng of cDNA template (used in triplicate) with QuantiFast SYBR Green master mix (Qiagen) with specific forward and reverse primers of the gene of interest for mRNA analysis and with a mix of forward specific primers of the mature miRNA and reverse universal qPCR reverse primer provided by the NCode VILO miRNA cDNA synthesis kit (Invitrogen). The primers are listed on Supplementary Table S1. The specificity of the PCR amplicon (size and product) and absence of primer-dimer were verified by melt-curve analysis using BioRad CFX manager software (Biorad). PCR conditions were as follows: 1 cycle of 95°C for 3 min followed by 40 cycles of 95°C for 10 s and 60°C for 60 s, with a final melt curve analysis step (heating the PCR mixture from 65 to 95°C by 0.5°C every 5 s). Samples were normalized to the 6 S rRNA level for quantification of the mRNA transcript and with the snU6 level for quantification of mature miRNA. Finally the relative levels of expression of miRNA and mRNA were determined using the 2^−ΔΔCt^ method. To quantify plasmid DNA content in tissues, absolute quantitative genomic PCR was performed as previously described (28). Briefly, 50 ng of extracted genomic DNA samples were used in each quantitative PCR reaction performed in triplicate using CymR primers. The absolute value of RILES plasmids DNA amount in tissues was determined using a standard curve performed with 5 µl of several dilutions of known concentrations of RILES plasmids in presence of CymR primers.

### Animal experiments

Animal housing and procedures were carried out according to the guidelines of the French Ministry of Agriculture for experiments with laboratory animals (Law 87848, C. Pichon accreditation). Female, 8-week-old outbred Swiss mice (BALB/c genetic background) and athymic nude mice were obtained from Harlam (France). Intramuscular injections of expression plasmids were performed as previously described: 8 µg expression plasmid or 2 µg inducible expression plasmid plus 6 µg pQE30 plasmid formulated with the amphiphilic block copolymer 704 (29) were administered into the tibialis anterior muscles of mice. Hydrodynamic injections were prepared in a saline physiological buffer corresponding to 10% body volume of the mouse and were administered over a 5-s period into the tail vein of mice (30). Atrophy was induced in female nude mice (Harlam) aged 8 weeks by sciatic nerve transaction as previously described (31). The animals were isofluorane-anesthetized and the left hindlimb (at the level of the femur) of the mice was exposed before making a small incision to isolate the sciatic nerve. Then a segment of 5 mm in length of the sciatic nerve was cut and carefully removed. Muscle and skin incisions were subsequently closed using 4-0 sutures.

### Bioluminescence imaging

Bioluminescence imaging was performed using either the NightOWL I LB (Berthold, Bad Wildbad Germany) or the IVIS Lumina II (PerkinElmer) imaging scanner coupled to the Indigo Software (Berthold) or the Living Image Software (PerkinElmer), respectively. Briefly, 2 mg of *in vivo* luciferase substrate (beetle luciferin substrate, Promega) were injected intraperitoneally in each mouse. Five minutes later, the mice were isofluorane-anesthetized and scanned. The abdominal cavity and the lower legs of mice were shaved once a week to allow accurate collection of bioluminescence signals. Light emissions were quantified from regions of interest (ROI) drawn manually and quantified using the imaging software. The sensitivity of the imaging scanner was tested weekly with commercially available positive sources of bioluminescence.

### Statistical analysis

Statistical analysis was performed using Prism (GraphPad software). Dual comparisons were made by the two-tailed student *t*-test. *P < 0.05; **P < 0.01 were considered as significant.

## RESULTS

### Molecular construct and design

**Figure 1.**
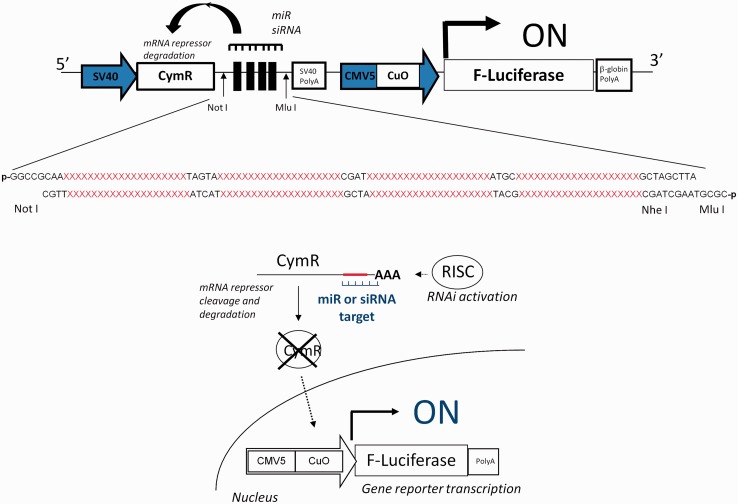
Schematic representation of the RILES method. When present in cells, target miRNA or siRNA binds to the four complementary-block sequences located in the 3′UTR of the CymR repressor transcript and activates the RNAi silencing complex (RISC) machinery. The CymR mRNA is then cleaved and degraded, resulting in lack of repressor production. The luciferase expression system is thus switched-ON, generating a positive bioluminescence signal.

We constructed a Cumate gene-switch expression system by assembling into a single plasmid unit the expression cassette encoding for the CymR repressor driven by the SV40 promoter and the inducible expression cassette encoding for the luciferase gene reporter driven by the CMV5(Cuo) inducible promoter (Figure 1). This dual expression system was called RNAi-Inducible Luciferase Expression System and denoted RILES. A multiple cloning site was then subcloned in the 3′ location of the CymR repressor cDNA to insert, on-demand, a block of four perfect-match complementary sequences to RNAi molecules such as siRNA or miRNA. The system is designed in such a way that it is the RNAi molecule that induces the expression of the luciferase gene. For instance, if a miRNA of interest is not expressed in the cell, the CymR transcript is produced as well as the CymR repressor protein. Consequently, the repressor protein binds to the Cuo operator and blocks transcription of the luciferase gene. Under this configuration, RILES is switched-OFF and no bioluminescence activity is expected. In contrast, if the miRNA of interest is expressed in the cell, it binds to the 3′UTR region of the CymR transcript, resulting in activation of the endogenous RNAi machinery. Consequently, no CymR repressor protein is produced enabling the luciferase gene reporter to be transcribed. Therefore, under this configuration, the RILES system is switched-ON and bioluminescence activity is detected.

### *In vitro* validation studies

**Figure 2.**
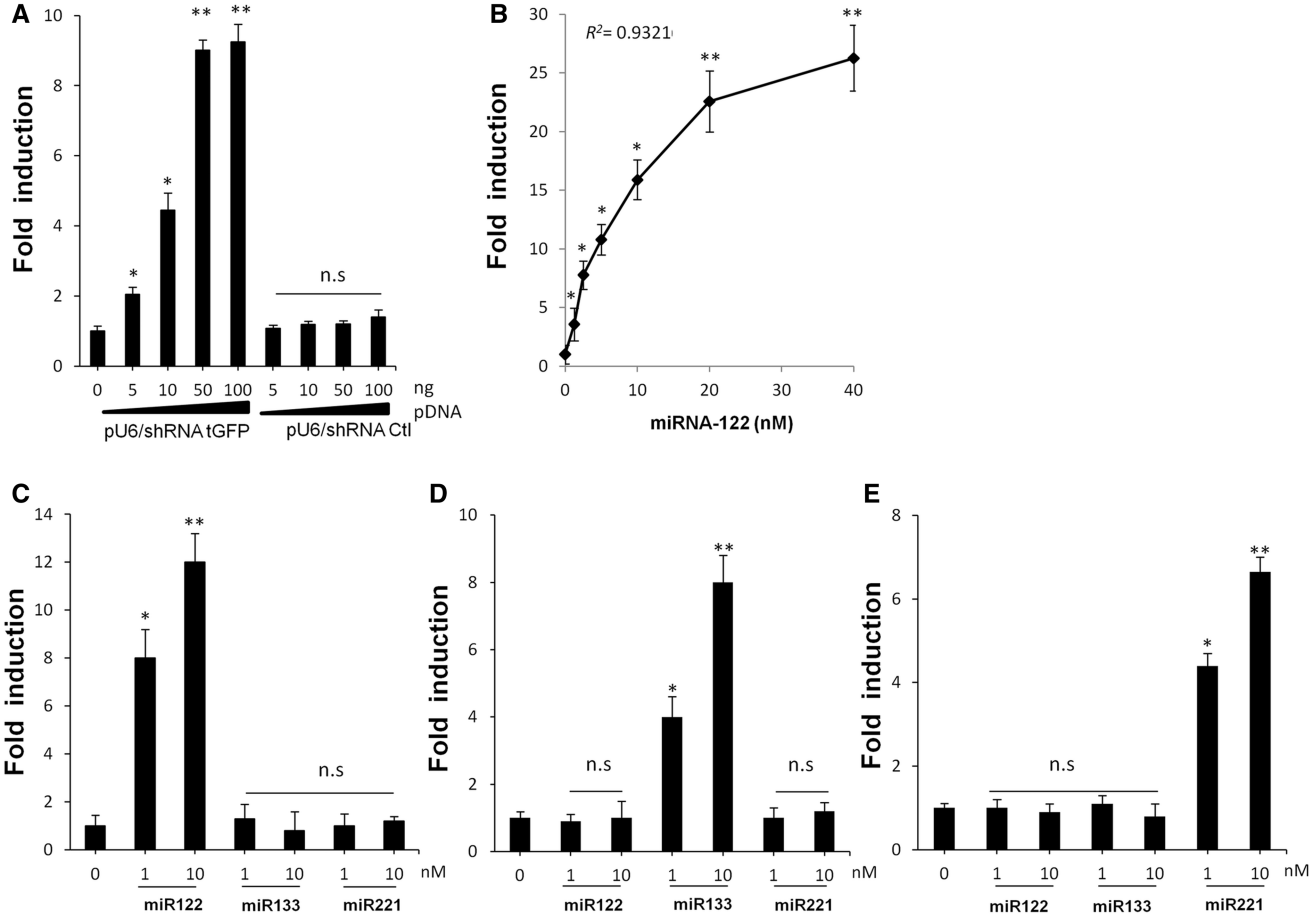
Luciferase expression in HEK 293 cells transfected with several RILES plasmids. Dose–response study of luciferase expression in HEK 293 cells transfected with (**A**) pRILES/siRNA tGFP T or (**B**) pRILES/122T in presence of (A) increasing amounts of siRNA tGFP (pU6/shRNA tGFP) and control siRNA (pU6/shRNA Ctl) or (B) increasing concentrations of synthetic miRNA-122. Selective luciferase expression in HEK 293 cells transfected either with (**C**) pRILES/122T, (**D**) pRILES/133T or (**E**) pRILES/221T in the presence of two concentrations of synthetic miRNA-122, −133 and −221. Forty-eight hours after transfection, luciferase expression in cells was determined and expressed as fold induction relative to control cells transfected with the plasmids alone and set to the arbitral value of 1. Data shown are the mean ±SD of one representative experiment performed in triplicate and reproduced at least three times. Statistics by the two-tailed *t*-test, *P < 0.05; **P < 0.01, n.s (no statistically significant difference) compared to control cells.

We first performed a series of *in vitro* proof of principle experiments to evaluate the specificity and the sensitivity of RILES in response to exogenous expressed RNAi molecules such as siRNA molecules encoded by a shRNA plasmid and synthetic miRNAs. Several RILES plasmids were constructed (Supplementary Table 1) and denoted for example pRILES/siRNA tGFPT or pRILES/122T when the RNAi targeting cassette contained complementary sequences to detect the siRNA tGFP or miRNA-122, respectively. These two RILES plasmids, pRILES/siRNA tGFPT and pRILES/122T, were individually transfected in HEK 293 cells in presence of increasing amounts of the shRNA plasmid (Figure 2A) or synthetic precursor miRNA-122 (Figure 2B). Forty-eight hours later, the luciferase activity in cells was determined and expressed as relative fold of luciferase induction by normalizing the values to cells transfected with the pRILES plasmid alone. As shown in Figure 2A and B, increasing the amount of shRNA tGFP plasmid (Figure 2A) or synthetic miRNA-122 (Figure 2B) in pRILES transfected cells also increased the luciferase fold induction values. A maximum 9-fold (±0.3, *n* = 3, *P* ≤ *0.01*) luciferase induction was found in response to 50 ng shRNA tGFP plasmid (Figure 2A) and a maximum of 26-fold (±1.2, *n* = 3, *P* ≤ *0.01*) was detected in response to 40 nM of miRNA-122 (Figure 2B). In the latter, the luciferase fold induction was found well correlated (*R*^2 ^= 0.9321) with the concentration of miRNA ranging from 0 to 20 nM. Overall, our experiments indicated that RILES is able to detect 1 nM of synthetic precursor miRNA molecules in transfected cells; this may represent the detection limit of RILES. It is worth noting that when the second generation of synthetic miRNA molecules (miR Mimics) was used, the detection limit of RILES was as low as 0.3 nM (data not shown). To assess the specificity of RILES, the experiments were conducted with a control mismatch shRNA plasmid (Figure 2A) or with irrelevant miRNAs (Figure 2C–E). No significant luciferase induction was detected in these assays. In contrast luciferase induction was detected only in cells transfected with pRILES/122T, pRILES/133T and pRILES/221T in presence of the corresponding miRNA-122, miRNA-133 and miRNA-221 (Figure 2C–E). We also found that three different synthetic miRNAs investigated at the same concentration were all equally efficient in switching-ON the configuration of RILES and inducing the same level of luciferase gene expression (data not shown). We finally assessed whether RILES could distinguish two closely related miRNA sequences that differ by two nucleotides such as two members of the miRNA-200 family. Results indicated that the pRILES/200cT did not distinguish these two miRNA sequences (Supplementary Figure S1). Next we wanted to determine whether RILES could also monitor the expression pattern of endogenous expressed miRNAs from established cell lines. For this purpose, we exploited the fact that HUH7 and HLE cell lines express opposite levels of miRNA-122 (32) and miRNA-221 (33). These cell lines were transfected individually with the pRILES/122T and pRILES/221T, and luciferase induction was determined 3 days later by normalizing the luciferase values to those found in cells transfected with the control untargeted miRNA RILES plasmid (pRILES). As shown in Supplementary Figure S2, the luciferase induction pattern detected in these cells was found to be remarkably similar to the miRNA expression pattern measured by quantitative RT-PCR. MiRNA-122 and miRNA-221 were oppositely expressed in HUH7 and HLE cells, while miRNA-133 was not significantly detected. Similar specificity of data was also found in C2C12 myoblast cells differentiated in myotubes *in vitro* to induce expression of the muscle-specific miRNA-133 (34). Again data from quantitative RT-PCR and bioluminescence analysis indicated a similar miRNA expression pattern, i.e. expression of miRNA-133 and almost undetectable expression of miRNA-122 and -221 in differentiated C2C12 cells (Supplementary Figure S2). Remarkably, RILES was also found to be functional in primary hard-to-transfect cells. The luciferase fold induction in primary culture of human dermal fibroblast NHDF was similar to the endogenous expression pattern of miRNA detected by quantitative PCR (data not shown).

### *In vivo* monitoring of miRNA-122 expression in the liver

**Figure 3.**
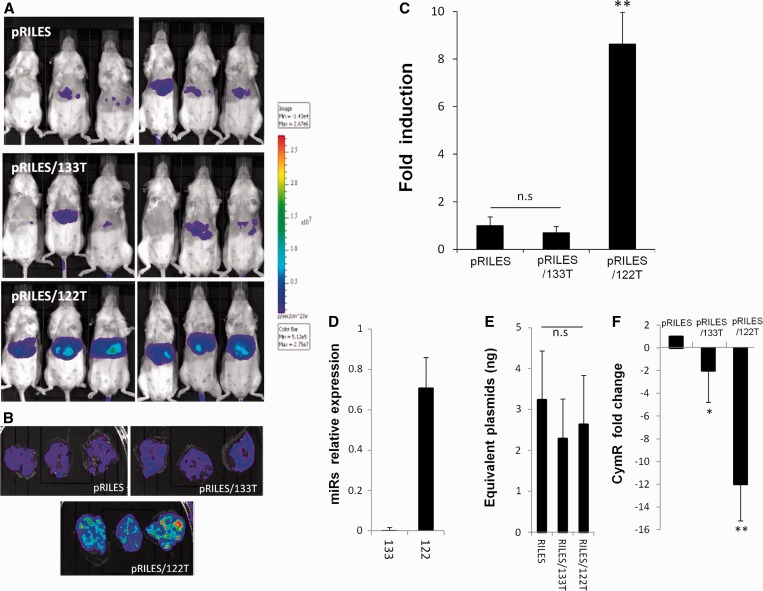
Noninvasive bioluminescence imaging of the liver-specific miRNA-122 in mice. Fifty micrograms of pRILES/122T and pRILES/133T were hydrodynamically injected in Swiss mice to transfect the liver. Negative control included the pRILES, not regulated by miRNA. Bioluminescence imaging was performed 3 days later and light emission quantified using ROIs covering (**A**) the whole abdominal cavity of the mice or (**B**) the liver of one representative mouse per group. (**C**) Quantitative bioluminescence values detected in mice described in A and expressed as luciferase induction relative to the control pRILES group of animals set arbitrarily to the value of 1. (**D**) Quantitative RT-PCR analysis of miRNA expression in the liver tissues of another group of mice. (**E**) Absolute quantification of plasmid content in the liver tissues of the mice described in B. (**F**) Quantitative RT-PCR analysis of CymR expression in the liver tissues of the mice described in B. Results are expressed as CymR fold change relative to control pRILES-tissues set arbitrarily to the value of 1. Error bars in C, mean ± SEM (*n* = 6) of one representative experiment repeated two times. Error bars in D, E, F mean ± SD (*n* = 3) of one representative experiment repeated at least three times. Statistics by the two-tailed *t*-test, *P < 0.05; **P < 0.01, n.s (no statistically significant difference) compared with the pRILES control group.

We then examined whether similar data could be collected through whole-body imaging of live-anesthetized animals and in real time. As miRNA-122 is exclusively expressed in the liver (32) and miRNA-133 is a muscle-tissue–specific miRNA (34), we enquired whether RILES would have the potential to discriminate the expression of these two miRNAs in the liver of the mice. To transfect the liver, we hydrodynamically injected (30) pRILES/122T and pRILES/133T and as control, the untargeted miRNA RILES plasmid (pRILES). Three days after administration, the mice were placed under a bioluminescence scanner to detect expression of the luciferase reporter gene. As shown in Figure 3A, a strong bioluminescent signal was detected in the abdominal area of the pRILES/122T-treated group of mice compared with the bioluminescent signals collected in the pRILES/133T- and the pRILES-treated groups, which both generated weak signals. The autopsy of one representative mouse per group of animals indicated that the liver was the source of emitted light (Figure 3B). Quantitative analysis showed a statistically significant 8.5-fold (±1.5, *n* = 3, *P* ≤ *0.01*) increase in luciferase activity in the liver of mice administered with pRILES/122T compared with the luciferase activity detected in the pRILES control group and normalized to the arbitral value of 1 (±0.5, *n* = 3) (Figure 3C). No statistical difference (*P* > 0.05) was found between the pRILES/133T and pRILES groups, indicating, as expected, that miRNA-133 was not expressed in the liver. To assess the specificity of our data, we compared the pattern of bioluminescence signals detected in mice with the expression pattern of miRNA detected by quantitative RT-PCR. Results indicated that these two methods generated similar data (Figure 3D versus 3C). Quantitative RT-PCR demonstrated that miRNA-122 is expressed in the liver in contrast to miRNA-133, which was almost undetectable in the liver samples. To demonstrate that the bioluminescence signal did not arise from an unspecific effect or from an uncontrolled dose of administration of the RILES plasmids, a quantitative genomic PCR was conducted to measure the absolute amount of RILES plasmids present in the liver. No statistically significant difference (*P* > 0.05) was found between the three groups of animals (Figure 3E), demonstrating that the bioluminescence signals arose rather from activation of the endogenous RNAi machinery. To fully validate this point, we conducted a relative quantitative RT-PCR analysis on the same tissue samples to evaluate the expression level of the CymR repressor transcript. The CymR transcript was found to be significantly 12-fold downregulated (±1.8, *n* = 6, *P* ≤ 0.01) in the liver tissues of the pRILES/122T-treated group compared with the pRILES control group (Figure 3F). A significant but less pronounced 2-fold (±1.6, *n* = 6, *P* < 0.05) downregulation of cymR transcript was found in the liver tissues of the pRILES/133T-treated group, whereas this group did not generate significant bioluminescence signals (Figure 3F versus 3C). These data indicate that the expression of miRNA-133 in the liver is not sufficient to repress a sufficient amount of CymR transcript to switch-ON the RILES in the liver of the mice.

### *In vivo* monitoring of myomiRNA expression in the tibialis anterior skeletal muscles

**Figure 4.**
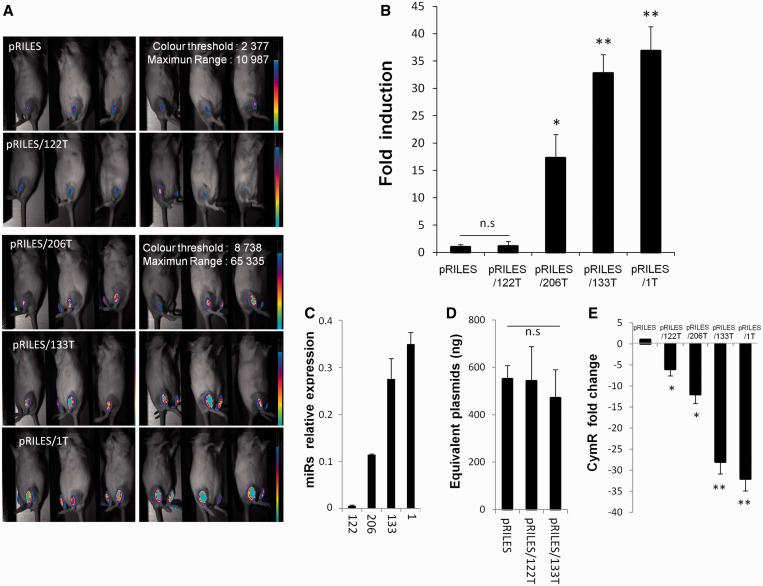
Noninvasive bioluminescence imaging of the muscle-specific myomiRs-206, −133 and −1 in mice. Eight micrograms of pRILES/122T, pRILES/133T, pRILES/1T and pRILES/206T were formulated with the 704 amphiphilic block copolymer and intramuscularly injected in the left and right tibialis anterior to transfect the skeletal muscles of Swiss mice. Negative control included the pRILES, not regulated by miRNA. Bioluminescence imaging was performed 6 days later and light emission quantified using ROIs covering the lower legs of the mice. (**A**) Representative bioluminescence images collected in the left lower legs of mice. (**B**) Quantitative bioluminescence values detected in the mice described in A and expressed as luciferase induction relative to the control pRILES group of animals set arbitrarily to the value of 1. (**C**) Quantitative RT-PCR analysis of myomiR expression detected in the tibialis anterior muscles of another group of mice. (**D)** Absolute quantification of plasmid content in the skeletal muscle of the mice described in A. (**E**) Quantitative RT-PCR analysis of CymR expression in the skeletal muscle of one representative scanned mouse described in A. Results are expressed as CymR fold change relative to control pRILES-tissues set arbitrarily to the value of 1. Error bars in B, mean ± SEM (*n* = 6) of one representative experiment repeated at least three times. Error bars in C, D, E, mean ± SD (*n* = 3) of one representative experiment repeated at least three times. Statistics by the two-tailed *t*-test, *P < 0.05; **P < 0.01 n.s (no statistically significant difference) compared with the pRILES control group.

Next, we wanted to evaluate the quantitative potential of RILES. We thus attempted to monitor the expression pattern of miRNA-1, miRNA-133 and miRNA-206 in the skeletal muscles of the anterior tibialis of the mice. These muscle-specific miRNAs and known to be differentially expressed in adult skeletal muscle tissue (34). To transfect the skeletal muscles efficiently the RILES plasmids were formulated with the amphiphilic block copolymer 704 (704) as described in (29) and then intramuscularly administered in the two tibialis anterior muscles of naive mice. Six days later (Figure 4A) strong bioluminescence signals were detected in the lower legs of mice administered with pRILES bearing the myomiRNA targeting cassette 1T, -133T and -206T. In contrast, low, almost undetectable bioluminescence signals were detected in the pRILES and pRILES/122T groups. Quantification of the bioluminescence signals indicated that the induction index of luciferase expression in the pRILES/1T-, pRILES/133T- and pRILES/206T-treated groups was, respectively, 37- (±4.4, *n* = 6, *P* < 0.01), 33- (±3.5, *n* = 6, *P* < 0.01) and 17- (±4.2, *n* = 6, *P* < 0.01) fold higher than the bioluminescence signal detected in the pRILES control group and normalized to the arbitral value of 1 (±0.4, *n* = 6) (Figure 4B). No statistical difference was found between the pRILES/122T and the pRILES group, suggesting that, in contrast to the liver, miRNA-122 is not expressed in the skeletal muscles. We then compared data generated using our method with those detected using quantitative RT-PCR. Remarkably, both methods provided similar data and conclusions (Figure 4B and C). MiRNA-122 was only faintly detected in the skeletal muscle, while miRNA-206, -133 and -1 showed an increasing expression level (Figure 4C versus 4B). The autopsy of one representative mouse per group indicated that all skeletal muscle tissues received the same amount of plasmids (Figure 4D), eliminating again the possibility that signals in mice could arise from a nonreproducible administration procedure of the plasmids *in vivo*. Immunohistochemical analysis indicated that 80% of the skeletal fibers of the pRILES/1T anterior tibialis tissues were stained by the luciferase antibody with an expected restricted localization to the skeletal muscle cells, which is in line with the unique cellular source of miRNA-1 expression in the anterior tibialis (Supplementary Figure S3), whereas no significant staining was detected in the pRILES and pRILES/122T muscle tissues. We also examined the expression level of CymR repressor transcript by quantitative RT-PCR. We found a significant reduction of CymR transcript in all skeletal muscle tissues investigated that was inversely correlated with the bioluminescence values (Figure 4E versus 4B). Indeed a mean of 32-fold (±2.5, *n* = 3, *P* < 0.01) of CymR downregulation was found in the pRILES/1T skeletal muscle tissues, which generated 37-fold (±4.4) more bioluminescence than pRILES control mice. A mean of 28-fold (±2.5, *n* = 3, *P* < 0.01) of CymR downregulation was found in the RILES/133T skeletal muscle tissues, which generated 33-fold (±3.5, *n* = 6) more bioluminescence than the pRILES control mice. Remarkably, an intermediate mean of 12-fold (±1.9, *n* = 3, *P* < 0.05) of CymR downregulation was found in the pRILES/206T skeletal muscle tissues, which generated an intermediate 17-fold (±4.2, *n* = 6) bioluminescence signal in mice compared with pRILES control mice (Figure 4E versus 4B). We also found a significant but lower 6-fold (±1.3, *n* = 6, *P* < 0.05) degradation of CymR transcript in the pRILES/122T-treated group, although miRNA-122 is weakly expressed in the skeletal muscles (Figure 4C) and unable to induce significant bioluminescence (Figure 4B). This indicated that the expression of miRNA-122 in the skeletal muscle is not sufficient to repress a sufficient amount of CymR transcript to switch-ON the RILES *in vivo*.

### Kinetics of miRNA expression in immunocompetent and immunodeficient mice

**Figure 5.**
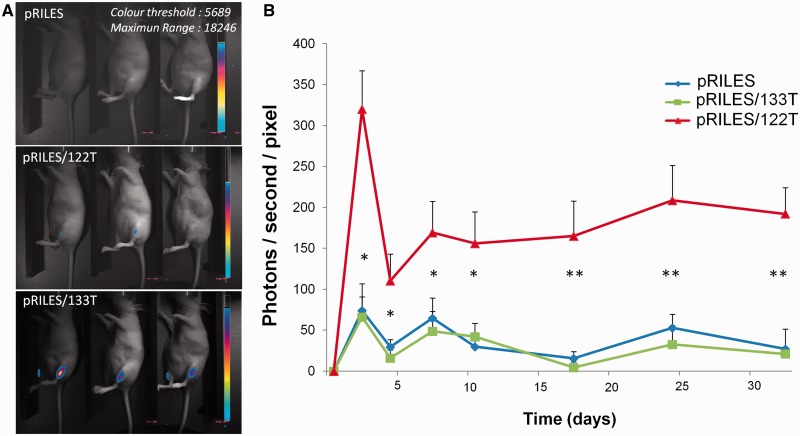
Kinetics of luciferase expression in the tibialis anterior muscle of immunodeficient mice. Two micrograms of pRILES/133T, pRILES/122T and control pRILES combined with 6 µg of the pQE30 empty expression plasmid were formulated with the 704 amphiphilic block copolymer and intramuscularly injected in the left and right tibialis anterior to transfect the skeletal muscles of nude mice. ROIs covering the lower legs of animals were drawn and light emission was quantified over time, from day 1 (before the intramuscular injection of RILES-derivates plasmids) to day 38, the end point of the assay. (**A**) Representative bioluminescence images collected at day 12 from three out of five mice per group. (**B**) Quantification of bioluminescence signals emitted from mice and plotted as a function of time. Error bars in B, mean ± SEM (*n* = 6) of one representative experiment repeated two times. Statistics by the two-tailed *t*-test, *P < 0.05; **P < 0.01 compared with the pRILES control group.

As miRNA-133 is constitutively expressed in the adult stage of skeletal muscles (34), we conducted a bioluminescence kinetic analysis of miRNA-133 expression in the tibialis anterior muscle of the mice. pRILES/133T, with pRILES/122T and pRILES as controls, was formulated with the 704 copolymer and then intramuscularly injected in the tibialis anterior muscle of mice. The bioluminescence signals emitted from the lower legs of animals were collected at several time points and expressed as the mean of bioluminescence as a function of time (day). As shown in Supplementary Figure S4, a peak of bioluminescence was detected for each group of mice 3 days after the intramuscular administration. Then a stable and constant bioluminescence signal ranging from day 5 to day 15 was observed irrespective of the group of mice. At this plateau, a statistically significant difference (*P* < 0.01) in luciferase expression was detected in the pRILES/133T versus pRILES/122T and pRILES groups of mice. However, after day 15, bioluminescence signals in the pRILES/133T group rapidly decreased to finally become undetectable at day 20. To determine whether this loss of bioluminescence signal may result from a nonspecific regulation of the inducible promoter, we re-administered the same amount of RILES derivated plasmids and collected the bioluminescence signals subsequently. The bioluminescence signals in mice were lower than those obtained after the first injection and were not statistically different between the various groups of mice (Supplementary Figure S4). We conducted similar experiments in immunodeficient mice to determine whether the loss of luciferase expression was finally related to the development of an immune response directed against either or both the bacterial origin of the CymR repressor or the luciferase protein. In these mice, we found (Figure 5) that the bioluminescence signals detected at the plateau of expression persisted over time and were stable for at least 34 days (end point of our experiment). These data indicate that using RILES, the expression of miRNAs can be monitored for a long period in immunodeficient mice.

### Dynamic monitoring of miRNA expression during atrophy development in immunodeficient mice

**Figure 6.**
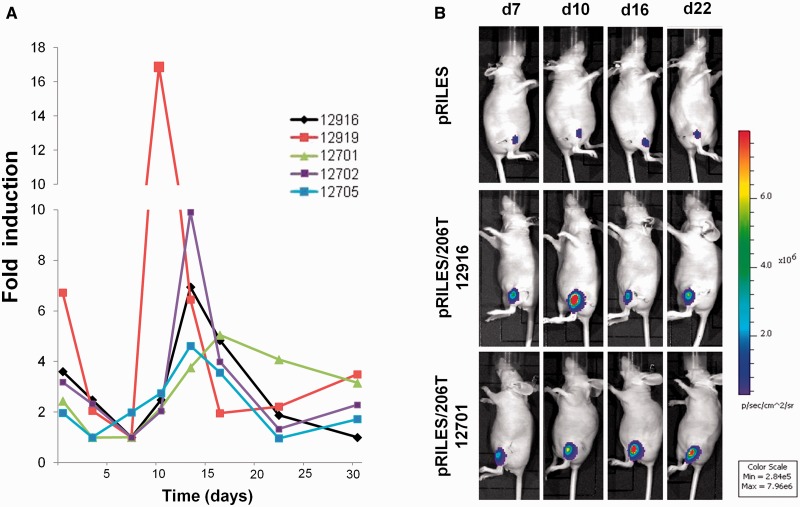
Real time monitoring of miRNA-206 regulation during development of skeletal muscle atrophy. Two micrograms of pRILES/206T combined with 6 µg of the pQE30 empty expression plasmid were formulated with the amphiphilic block polymer 704 and intramuscularly injected in the tibialis anterior to transfect the skeletal muscles of nude mice. Three days later (day 0), bioluminescence activity in the lower legs of mice was measured, and the left sciatic nerves were cut surgically to induce denervation and atrophy. Mice (*n* = 5) were thereafter scanned twice a week for the first 3 weeks and then once a week till day 35 (end point of our experiment). (**A**) Kinetic of miRNA-206 expression detected in each individual mouse by bioluminescence imaging. Results are expressed as relative fold of luciferase induction by normalizing the bioluminescence values to the minimal value found before the bioluminescence peak for each single mouse. (**B**) Representative bioluminescence images collected from one representative mouse from the pRILES group and two representative mice from the pRILES/206T group at days 7 (d7), 13 (d13), 22 (d22) and 30 (d30). The number of each mouse for identification during the longitudinal study is given.

To fully validate the potential value of’ RILES and propose a relevant application in the field of miRNA biology, we examined whether the system may be suitable to monitor the dynamic regulation of miRNAs under pathophysiological conditions. To assess this point, we used a muscular atrophy model obtained by complete section of the sciatic nerve of the mice. Recently, miRNAs were found or alleged to be involved in response to this injury (35). In a pilot experiment study, we found that among the three different myomiRs investigated by *in vivo* bioluminescence imaging, miRNA-206 was the most strongly regulated miRNA (data not shown). To, first, evaluate the regulation of luciferase expression during development of this chronic disease, characterized by significant metabolic changes (36–38), we injected in the tibialis anterior of the mice an expression plasmid containing the second expression cassette of RILES plasmid (CMV5/Cuo/F-luc, pRILES/Fluc) for constitutive expression of the luciferase gene reporter in the denervated skeletal muscle. As expected, the bioluminescence signals emitted from the control, not-atrophied-leg, injected with pRILES/F-Luc were stable for at least 35 days (Supplementary Figure S5A, red line and S5B, control), whereas results obtained from the pRILES/Fluc-denervated skeletal muscle resulted in a rapid loss (within 3 days) of the bioluminescence signal (Supplementary Figure S5A, blue line and S5B, section). From day 7, the signal remained almost stable for 35 days. This change in luciferase expression was paralleled by the rate of muscle-weight loss (Supplementary Figure S5C) and may be a consequence of a specific transcriptional program governing the expression of ubiquitin ligases, as described in (36,37). On this basis, we decided to determine the kinetic of miRNA-206 expression during the second phase of this chronic muscular disease, known as the adaptive response phase (37). pRILES/206T and pRILES were injected intramuscularly in the tibialis anterior muscle of mice, and 3 days later the sciatic nerve of the left leg was surgically sectioned. The mice were thereafter imaged twice a week for the first 2 weeks and then once a week for 3 weeks. Bioluminescence imaging of the atrophied legs of mice administered with the control, untargeted miRNA, pRILES showed a low and stable level of luciferase expression (mean of 3.12 × 10^6^ photons/s, ±4.63 × 10^3^, *n* = 5) during the whole kinetic course of the study (Supplementary Figure S6A). Quantitative analysis of pooled data from five animals administered with the pRILES/206T showed a much higher luciferase activity (mean of 4.26 × 10^7^ photons/s, ±9.3 × 10^6^, *n* = 5), which decreased 2-fold immediately after nerve section, probably in relation with the overall loss of metabolic activity documented in Supplementary Figure S5. Then, the luciferase activity reached a first plateau from days 3 to 7 and then increased quickly and peaked at day 13 before finally dropping at day 22 to the value of day 6 until day 30. In sharp contrast, the luciferase activity in the nonatrophied leg, intramuscularly injected with the pRILES/206T plasmid was constant during the whole course of the kinetic analysis (data not shown). In a separate experiment, miRNA-206 expression was determined by quantitative RT-PCR at days 6 (*n* = 5), 12 (*n* = 5), 19 (*n* = 5) and 27 (*n* = 5) after muscle denervation. Quantitative PCR data showed a similar pattern of miRNA-206 expression as detected by bioluminescence imaging (Supplementary Figure S6A) characterized by an increase of miRNA-206 expression detected from day 12 to day 19 and a decrease by day 27 after denervation (Supplementary Figure S6B). Both bioluminescence and quantitative RT-PCR data showed strong variability, and the only statistical significance detected by quantitative RT-PCR data was found by comparing day 6 with day 12, and day 12/19 with day 27. We then fully exploited the advantage of the noninvasive nature of the RILES method to determine the source of variability in each individual mouse. As shown in Figure 6, a strong heterogeneity in the level of miRNA-206 expression was observed between the mice although the kinetic of miRNA-206 expression exhibited a bell-shaped aspect with a maximum bioluminescence value bordered by two minimal values for all mice. We found that expression of miRNA-206 peaked at three different time points, either at day 13 (3 out of 5 mice) or day 10 (1 out of 5 mice) or even later, at day 18 (1 out of 5 mice) (Figure 6A). To quantify the fold induction change in miRNA-206 expression, we normalized the bioluminescence values monitored for each mouse to the minimal value detected at day 6 after denervation and set to the arbitral value of 1. Data from quantitative PCR (supplementary Figure S6B) and *in vivo* bioluminescence experiments (Figure 6A) indicated that at day 6, the expression of miRNA-206 was indeed at its lowest value. Normalized data indicated that the luciferase induction of miRNA-206 regulation ranged heterogeneously from a minimal value of 2-fold to a maximum value of 17-fold. The amplitude of miRNA-206 regulation was also found to be dependent on the mouse, extending from a minimum period of 4 days to a maximum period of 20 days. Similar trends of miRNA-206 expression were found in other sets of experiments performed. We invariably detected a major peak of miRNA-206 expression at day 13 (3 out of 5 mice) and two additional peaks detected at day 10 (1 out of 5 mice) and 16 (1 out of 5 mice) (data not shown). In addition, the miRNA-206 induction level as well as the amplitude of expression was variable between the mice, ranging from 2- to 12-fold induction and to 4–16 days of time of regulation (data not shown). Altogether these data demonstrate the great potential of the RILES method to generate relevant information about miRNA regulation and emphasize the importance of the temporal dimension of miRNA analysis during the development of biological processes.

## DISCUSSION

Here, we described a novel method, RILES, to monitor, in real time and at the whole-body-scale of live animals, the dynamic expression pattern of miRNAs under both physiological and physiopathological conditions. Using RILES, we established for the first time in mice, the expression kinetic of a miRNA in an animal disease model during a period of 30 days. Our strategy is based on the use of the cumate gene-switch system (24) in which the CuO, an operator DNA-binding sequence of the CymR protein repressor, is positioned between the luciferase reporter gene and its promoter. Therefore, when the miRNA is functional, the CymR repressor is no longer produced and the luciferase reporter gene is then expressed. Thus, a fundamental molecular mechanism integrated in our system is that RNAi molecules (siRNA, miRNA, shRNA) have the capacity of specific inducers. This is different from the traditional approach where the ‘ON/OFF’ configuration of the expression system is controlled by the addition of exogenous inducers such as cumate, tetracycline or doxycycline for instance (26). All of the regulatory elements for this system to function were assembled in a single plasmid to easily transfect mammalian cells for *in vitro* studies or for *in vivo* bioluminescence studies. We validated the method *in vitro* using a panel of cell lines, *in vivo* in normal organs (skeletal muscles and liver), and finally in a mouse model of muscular atrophy. We demonstrated, in addition, that RILES is suited to the longitudinal study of miRNA expression in both physiological and pathophysiological organs without repetitive administration of RILES. Using a model of muscular atrophy, we were able to determine the kinetic of miRNA-206 expression during the muscle regeneration phase of muscle atrophy (36,37). The bioluminescence data indicated that the expression of miRNA-206 is individual-dependent, finely regulated in a time-dependent manner and characterized by individual heterogeneity during development of the pathology. When compared with data generated from conventional quantitative RT-PCR, miRNA-206 expression was also found to be overexpressed but its expression remained constant for 7 days before returning to the basal level. This discrepancy between the two approaches is explained by the invasive nature of the quantitative RT-PCR method that generates a set of information from a heterogeneous population collected at different time points. This invasive method is costly in terms of animal numbers and more importantly lacks temporal resolution at the individual level. Hence, conventional invasive methods undervalue the crucial significance of the temporal regulation of miRNA expression in response to biological processes (6–10). In contrast, because RILES is suited to a longitudinal study, sharper insights into miRNA regulation can be gathered, providing a novel dimension of miRNA expression analysis. These information are obviously relevant in the field of miRNA biology and more particularly in the field of muscle regeneration, which is currently the focus of intensive research (38,39). In addition, these data also have some therapeutic value. They indicate that underestimating the expression kinetics of miRNA-206 and the heterogeneity between individuals might reduce the benefit of a miRNA-based therapeutic approach aiming at restoring (replacement therapy) or reducing (antagonist therapy) the expression of miRNA-206 for the treatment of muscular disease as proposed in (39,40).

Our RILES method has additional advantages over current approaches devoted to the *in vivo* monitoring of miRNA expression. First, RILES is a positive-molecular-monitoring system that generates bioluminescence signals when the target miRNA is expressed in cells. This approach is more reliable than the negative-molecular-monitoring approach (15,16) where expression of miRNA is indicated by a reduction in the bioluminescence signal. Second, RILES is a sensitive method particularly well adapted to *in vivo* functional studies in small animals. Bioluminescence emission catalyzed by the enzymatic luciferase reaction is extremely efficient with a quantum yield of ∼88%, with low or no background and remains one of the most reliable reporter systems used to quantify gene expression in mammalian cells *in vivo* (41–43). However, environmental factors, such as oxygen, ATP, temperature and pH change have been shown to impact on the catalytic activity of the luciferase enzyme (44). In this case, the luciferase gene in RILES can be substituted by other optical reporter genes as the Tomato (45) or isotopic reporter genes such as the sodium/iodide Symporter (NIS) for instance (46). Third, RILES does not require a complicated procedure to generate different expression plasmids to monitor miRNAs. We designed an easily interchangeable miRNA T cassette that can be straightforwardly manipulated and used in any laboratory skilled in standard molecular biology techniques and equipped with standard *in vitro* and *in vivo* bioluminescence equipment. This contrasts with the multistep procedure and handling required to synthesize and label molecular fluorescent probes (47) that also are well known to have a low signal-to-background ratio when used *in vivo* (42). Again when considering fluorescent molecular beacons as a positive miRNA monitoring system (17,18), careful design of the probes needs to be performed first *in cellulo* before being validated *in vitro*, and this procedure has to be repeated for each miRNA of interest (48). However, the use of an expression plasmid to monitor the expression of miRNA also has some limitations. First, the long-term expression of a miRNA targeting sequence (miRNA T) in transfected cells might compete with the endogenous mRNA targets of the miRNA of interest. As a result, RILES might interfere with the biological process studied. Other studies have addressed such issues (49–51) and demonstrated that a saturable effect of the miRNA T cassette in cells is found only when the expression of transgenes bearing the miRNA targeting sequence is driven by a strong promoter. We anticipated this point and used the weak SV40 promoter to drive expression of the engineered CymR transcript. In addition, we did not find any statistical difference in terms of miRNA-206 expression between RILES-transfected tibialis muscle and not-transfected control muscle (data not shown). This indicates that the fraction of miRNA-206 bound to the CymR repressor transcript is not compensated by overexpression of miRNA-206 and strongly suggests that RILES is minimally interfering in cells. Second, when a long term monitoring study of miRNA expression has to be performed, RILES has a restricted application to immunodeficient mice. Although we did not demonstrate the presence of an immunological response developed against the prokaryotic origin of the CymR repressor molecule, it is likely that CymR is immunogenic in immunocompetent mice as already demonstrated with other prokaryotic-inducible expression systems (52,53). The use of transgenic animals bearing RILES in their genomes might overcome this limitation and will provide, in the meantime, a reliable tool to monitor the expression of miRNAs during embryonic development. Finally, as for most of the inducible expression systems described, promoter leakiness in the absence of inducer is a drawback (26). We did find some basal expression of the luciferase gene in the absence of RNAi molecules but the leakiness was low and not a limitation for our study, as evidenced by our results. It is worth noting that in HEK 293 cells, the time frame for pRILES to be accurately switched-OFF and consequently for the luciferase basal expression level to be at its minimal value is rapid, i.e. 12 h after transfection (Supplementary Figure S6). The observation that, at this same time point, the pRILES/122T is adequately switched-ON in the same cells transfected with synthetic miRNA-122 indicates that the delay for the RNAi machinery to suppress expression of CymR protein is also remarkably fast (Supplementary Figure S7). This supports the notion that using RILES, it might be possible to monitor changes in miRNA expression for periods at least equal to or above 12 h.

Overall, our data provide further compelling evidence of the great potential of the endogenous RNAi machinery to control the expression of a transgene in a sequence-specific manner. Indeed, miRNA-target sequences subcloned in the 3′UTR region of a transgene cassette have been shown to be a reliable tool to restrict transgene expression in specific cell types and lineages or to differentiate states (54–56). In all the systems currently used, the endogenous RNAi machinery is used to repress expression of the transgene (e.g. reporter gene, therapeutic gene, viral gene) (56). The success of this approach was proved by the experimental evidence that de-targeting a transgene expression in hematopoietic cells, using target sequences of hematopoietic lineage-specific miRNA-142.3, enabled stable and long-lasting transgene expression in mice without inducing an immune response, overcoming one of the most current issues in gene therapy (57). With RILES, the expression of the transgene is not suppressed by the endogenous RNAi machinery, but rather induced. Therefore, RILES can be used as a novel method to program the expression of transgenes in any targeted cells as long as a specific miRNA is expressed in these cells. We provided evidence that this programming approach is as robust, specific and tight as the de-targeting approach. The transgene expression, e.g. the luciferase reporter gene, was found to be tightly controlled by tissue-specific miRNAs, such as miRNA-122 in the liver and myomiRs-206, -1 and -133 in the skeletal muscles. We demonstrated that the amplitude of luciferase expression was correlated with the amount of miRNA expressed exogenously and endogenously and specific to the target miRNA sequence. We also demonstrated that expression of the luciferase protein was spatially restricted to skeletal muscle cells when the RILES plasmid was customized to be responsive to the muscle-specific miRNA-1. We finally provide the molecular evidence that RILES is tightly controlled by the endogenous RNAi machinery as expression of the luciferase reporter gene was found to be inversely correlated with the rate of repressor CymR transcript degradation detected by quantitative PCR. It is worth noting that another advantage of an RNAi-inducible expression system such as RILES is that such systems will overcome the low *in vivo* pharmacokinetics of exogenous inducers in small animals and in humans (26,58) that have compromised the stringent modulation of transgene expression in gene therapy (26). However, the immunogenicity of the prokaryotic form of the repressor is currently an important issue that requires further optimization to be applicable in immunocompetent individuals. Nevertheless, we envision that in addition to the application of RILES in the field of miRNA biology, RILES may serve as a platform for the development of a novel generation of controllable gene expression systems with potential research and therapeutic applications (59). In line with this perspective, a recent work by Naldini and his collaborators (60) demonstrated that lentiviral vectors encoding for the tTR-KRAB or the tTR repressor protein, placed under the control of hematopoietic lineage-specific miRNAs, were found to be a reliable method to positively identify and select *in vitro* a subset of hematopoietic stem and progenitor cell populations. When transplanted into mice, these selected cell populations were able to repopulate their respective spleen and bone marrow niches. Remarkably, the expression of the reporter gene within the niche was found to be consistent with the pattern of activity of the miRNAs expressed by the different types of implanted stem cells. These results are of importance as they suggest that it might be possible to transfer the expression of a therapeutic transgene to a subset of well-defined differentiated cell populations originated from transplanted stem cells *in vivo*. This work provides experimental evidence that programming the expression of a therapeutic transgene in targeted cells might be possible according to the differential expression pattern of miRNA.

In summary, we have provided here a complete proof of principle study and demonstrated that the use of an inducible expression system placed under the control of the endogenous RNAi machinery is a robust and reliable method to induce the expression of a transgene in target cells. We have demonstrated that when a sensitive imaging gene reporter is used as transgene, this system offers the possibility to study the dynamic regulation of miRNA in physiological and pathophysiological contexts. Because RILES offers a temporal analysis of the expression of miRNAs at the individual level, this method enables to collect more relevant biological information than does a conventional quantitative RT-PCR approach. Therefore, RILES has applications in the field of miRNA biology and may also represent a novel method to program the expression of therapeutic transgenes (cDNA, shRNA, miRNA mimic, antagomiRNA, RNAi sponge, etc) in specific target cells, with possible applications in the field of gene and cell therapy.

## SUPPLEMENTARY DATA

Supplementary Data are available at NAR Online.

## FUNDING

La Ligue Contre le Cancer du Loiret (to P.B.); Vaincre la Mucoviscidose, ANR and Plan Cancer 2009–2013 (to G.V.); La Région Centre, Ph.D funding (to S.E.). Funding for open access charge: La Ligue Contre le Cancer du Loiret Région centre.

*Conflict of interest statement*. None declared.

## Supplementary Material

Supplementary Data
