# Supplementary material for: RILES, a novel method for temporal analysis of the in vivo regulation of miRNA expression
Source: Nucleic Acids Res. 2013 Sep 5;41(20):e192. doi: 10.1093/nar/gkt797 (PMC3814383; doi:10.1093/nar/gkt797)
Supplement: Supplementary Data [file supp_41_20_e192__index.html]

RILES, a novel method for temporal analysis of the in vivo regulation of miRNA expression — RILES, a novel method for temporal analysis of the in vivo regulation of miRNA expression — Supplementary Data 

# RILES, a novel method for temporal analysis of the *in vivo* regulation of miRNA expression

## Supplementary Data

files

**Files in this Data Supplement:**

- Supplementary Data - pdf file
