# Supplementary material for: RILES, a novel method for temporal analysis of the in vivo regulation of miRNA expression
Source: Nucleic Acids Res. 2013 Sep 5;41(20):e192. doi: 10.1093/nar/gkt797 (PMC3814383; doi:10.1093/nar/gkt797)
Supplement: Supplementary Data [file supp_gkt797_nar-00889-met-g-2013-File008.pdf]

SUPPLEMENTARY DATA

Supplementary Table 1

**RILES plasmids**	**Name**	**RNAi Targeting sequence subcloned into 3'-untranslated region of CymR transcript**
RILES/CMV5(Cuo)/CymR/ F-Luc	pRILES	No RNAi targeting sequence
RILES/CMV5(Cuo)/CymR/ 4xsiRNA.tGFPT/F-Luc	pRILES /siRNA.tGFPT	GGCCGCAA**CGTGATCTTCACCGACAAGAT**TAGTA**CGTGATCTTCACCGACAAGAT**CGAT**CGTGATCTTCACCGACAAGAT**ATGC**CGTGATCTTCACCGACAAGAT**GCTAGCTTA
RILES/CMV5(Cuo)/CymR/ 4x122T/F-Luc	pRILES/122T	GGCCGCAA**CAAACACCATTGTCACACTCCAT**AGTA**CAAACACCATTGTCACACTCCA**CGAT**CAAACACCATTGTCACACTCCA**ATGC**CAAACACCATTGTCACACTCCA**GCTAGCTTA
RILES/CMV5(Cuo)/CymR/ 4x133T/F-Luc	pRILES/133T	GGCCGCAA**ACAGCTGGTTGAAGGGGACCAA**TAGTA**ACAGCTGGTTGAAGGGGACCAA**CGAT**ACAGCTGGTTGAAGGGGACCAA**ATGC**ACAGCTGGTTGAAGGGGACCAA**GCTAGCTTA
RILES/CMV5(Cuo)/CymR/ 4x1T/F-Luc	pRILES/1T	GGCCGCAA**TACATACTTCTTTACATTCCA**TAGTA**TACATACTTCTTTACATTCCA**CGAT**TACATACTTCTTTACATTCCA**ATGC**TACATACTTCTTTACATTCCA**GCTAGCTTA
RILES/CMV5(Cuo)/CymR/ 4x221T/F-Luc	pRILES/221T	GGCCGCAA**GAAACCCAGCAGACAATGTAGCT**TAGTA**GAAACCCAGCAGACAATGTAGCT**CGAT**GAAACCCAGCAGACAATGTAGCT**ATGC**GAAACCCAGCAGACAATGTAGCT**GCTAGCTTA
RILES/CMV5(Cuo)/CymR/ 4x206T/F-Luc	pRILES/206T	GGCCGCAA**CCACACACTTCCTTACATTCCA**TAGTA**CCACACACTTCCTTACATTCCA**CGAT**CCACACACTTCCTTACATTCCA**ATGC**CCACACACTTCCTTACATTCCA**GCTAGCTTA

Bold and underlined : the four-block sequence complementary to RNAi molecules

List of primers used:

For mRNA analysis

CymR Forward: 5'-AAGCTTGAGCTTCTGCTTGC-3'
CymR Reverse: 5'-GCTGAATACCCTCGCGTAAC-3'
6S Forward: 5'-CCAAGCTTATTCAGCGTCTTGTTACTCC-3'
6S Reverse: 5'-CCCTCGAGTCCTTCATTCTCTTGGC-3'

For miR analysis

miR-122: 5'-TGGAGTGTGACAATGGTGTTT-3'
miR-1: 5'-CCGGTGGAATGTAAAGAAGTATGTAT-3'
miR-133a: 5'-GTCCCCTTCAACCAGCTGAA-3'
miR-206: 5'-TGGAATGTAAGGAAGTGTGTGG-3'
U6: 5'-CGCAAGGATGACACGCAAATTC-3'

Supplementary Figure Legends

Supplementary Figure 1. Luciferase expression in HEK 293 cells transfected with pRILES/200cT and two members of the miRNA-200 family. HEK 293 cells were transfected with pRILES/200cT in presence of two different concentrations of synthetic miRNA mimics 200c-3p or 200b-3p. Two days later, luciferase expression was determined and expressed as luciferase induction relative to control cells transfected with the pRILES/200cT alone and set to the arbitral value of 1. Data shown are the mean +/- SD of three different experiments performed in triplicate. Statistics by the two-tailed t-test, *n.s* no statistically significant difference (*n.s*) between cells transfected with miRNA-200c-3p and miRNA-200b-3p.

Supplementary Figure 2. Correlation between luciferase activity and endogenous miR expression. (**A**) HuH7, (**B**) HLE hepatocarcinoma cell lines and (**C**) C2C12 differentiated cells were transfected individually with pRILES/133T, pRILES/122T, pRILES/221T and as control pRILES, not regulated by miR. Three days later, luciferase expression was determined and expressed as luciferase induction relative to cells transfected with the control pRILES. *Inset :* histograms representing the quantitative RT-PCR analysis of miR expression in the cells. Data shown are the mean +/- SD of one representative experiment performed in triplicate and reproduced at least three times. Statistics by the two-tailed t-test, * $p < 0.05$ compared with the pRILES control-transfected cells.

Supplementary Figure 3. Immunohistochemical detection of luciferase expression in the skeletal muscle tissues of mice injected intramuscularly with (**A**) pRILES, (**B**) pRILES/122T and (**C**) pRILES/1T. One representative mouse per group from Fig 4 was sacrificed and the tibialis anterior muscle harvested and stained with a specific luciferase antibody. Pictures shown are representative of a staining performed from at least ten serial sectioned tissues. Scale bar: 200 µM.

Supplementary Figure 4. Kinetics of luciferase expression in the tibialis anterior muscle of immunocompetent mice. 2 µg of pRILES/133T or pRILES/122T combined with 6 µg of the pQE30 empty expression plasmid were formulated with the 704 amphiphilic block copolymer and intramuscularly injected in the tibialis anterior to transfect the skeletal muscle of Swiss mice. Negative control includes the pRILES, not regulated by miR. Regions-of-interest covering the lower legs of animals were drawn and light emission was quantified over time, from day 1 (before the intramuscular injection of plasmids) to day 38 (endpoint of the assay). **A**) Representative bioluminescence images collected at day 12 from three out of five mice per group of animals. **B**) Quantification of bioluminescence signals emitted from mice and plotted as a function of time. *Arrow:* on day 25 when bioluminescence signals were lost, plasmids were again administered and the luciferase expression was determined two, four and six days later. Error bars in B, mean +/- SEM (n = 6) of one representative experiment repeated at least two times. Statistics by the two-tailed t-test, * $p < 0.05$; ** $p < 0.01$ compared with the PKDP control group.

Supplementary Figure 5. Bioluminescence imaging of the luciferase expression in a mouse model of muscular atrophy. The second expression cassette encoding for the luciferase gene reporter

(CMV5/Cuo/F-luc, RILES/F-Luc) was subcloned in an empty plasmid to generate a constitutive luciferase-expressing plasmid with the same backbone as the pRILES. 2 µg of pRILES/F-Luc combined with 6 µg of the pQE30 empty expression plasmid were formulated with the 704 amphiphilic block copolymer and intramuscularly injected in the tibialis anterior muscles of nude mice. Three days later, bioluminescence imaging was performed and then the left sciatic nerves of mice were cut surgically to induce muscular atrophy. Luciferase activity was measured on the left (sciatic nerve cut, n = 3) and right legs (control leg, n = 3) of each animal and plotted as a function of time. **A**) Kinetics of luciferase expression in the atrophied legs (blue line) compared to normal counterpart legs (red line). The data show that atrophy leads to a dramatic decrease in luciferase activity, starting immediately after section and reaching a plateau by day 7. **B**) Representative images of one out of three mice per group, scanned at days 3, 15 and 22. Top panel: control leg, lower panel: atrophied leg. **C**) Parallel between the loss of luciferase activity and the weight of denervated tibialis anterior muscles. A total of 18 immunocompetent mice (3 mice for each time point) were treated as described above and scanned at the indicated time. Immediately after, three random mice per group were sacrificed and the tibialis anterior muscles collected and weighed. Values are expressed as mean +/- SEM of photons/second (n = 3) *versus* mean +/- SD (n = 3) of muscle weight expressed in mg.

Supplementary Figure 6. Comparative study between the mean of bioluminescence values detected using RILES in live mice and data generated from quantitative RT-PCR. 2 µg of pRILES/206T (blue line, n= 3) or control pRILES (red line, n= 3) combined with 6 µg of the pQE30 empty expression plasmid were formulated with the 704 amphiphilic block polymer and intramuscularly injected in the left tibialis anterior muscles of nude mice. Three days later (day zero), bioluminescence activity was determined and the left sciatic nerve was cut surgically to induce denervation and atrophy. The mice were thereafter scanned twice a week for the first three weeks and then once a week for 32 days (end-point of our experiment). **A)** Mean of bioluminescence values quantified in pRILES/206T (blue line) and control pRILES (red line)-administered animals. **B)** Quantitative RT-PCR analysis of miR-206 expression detected in the tibialis anterior muscles of another group of mice. The left sciatic nerves of a total of 20 mice were sectioned and the tibialis muscles collected at day 6 (A6, n = 5), day 12 (A12, n = 5), day 18 (A18, n = 5) and day 27 (A27, n = 5) after denervation. Error bars in A, mean +/- SEM (n = 3) of one representative experiment repeated two times. Error bars in C, mean +/- SD (n = 3) of one representative experiment repeated two times. Statistics by the two-tailed t-test, * $p < 0.05$; compared to the bioluminescence value detected in *A* at day 6 and to the relative miR-206 expression detected in *B* at day 6.

Supplementary Figure 7. Kinetics of luciferase expression in HEK 293 cells transfected with RILES plasmids. The cells were transfected individually with pRILES/122T, pRILES/133T and control pRILES in presence of 10 nM of synthetic precursor miRNA-122. Four hours post transfection (T0), bioluminescence activity was measured using a bioluminometer and later monitored over time at the indicated time. Data are the mean +/- SD of one representative experiment performed in triplicate and reproduced two times. Statistics by the two-tailed t-test, * $p < 0.05$; ** $p < 0.01$, compared to control

cells. No statistical differences between the pRILES-transfected cells were found at an early time point (e.g 6 hours).

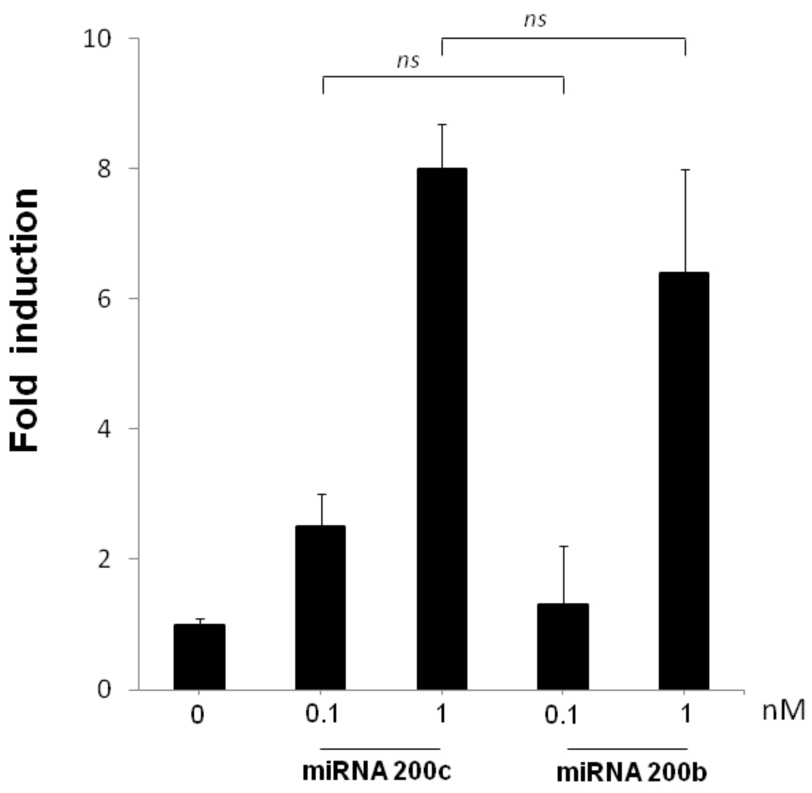
ns
ns
10
8
6
4
2
0
Fold induction
0
0.1
1
0.1
1
nM
miRNA 200c
miRNA 200b

Figure S1

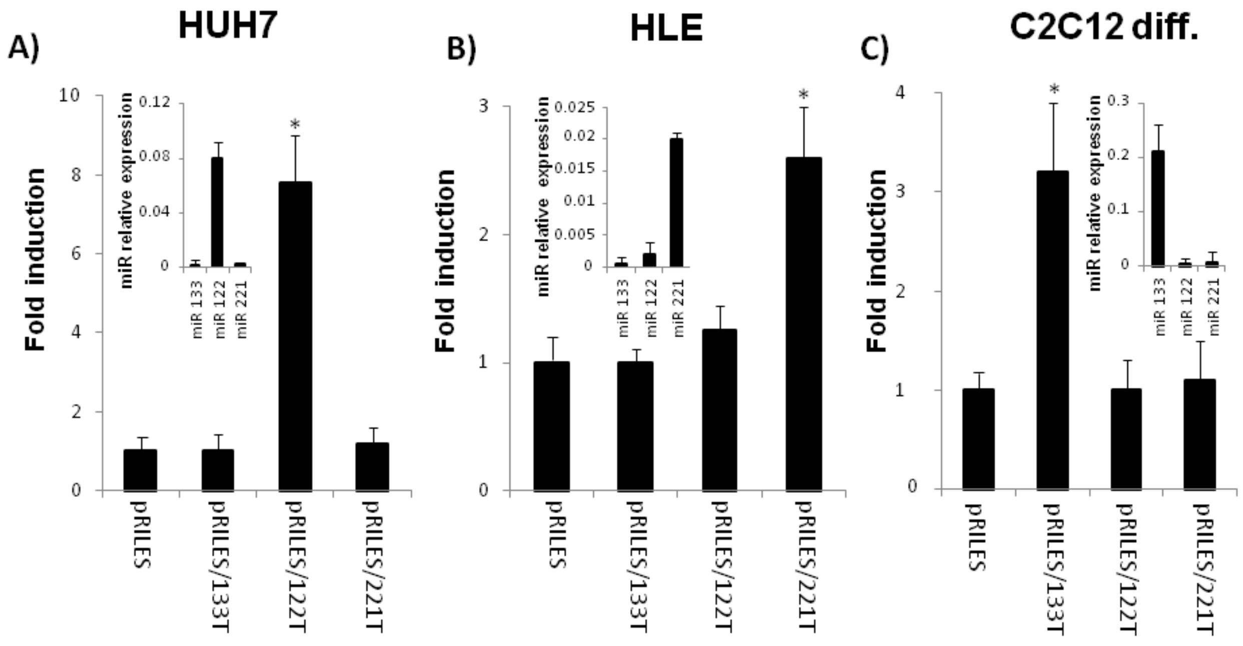

A)
HUH7
B)
HLE
C)
C2C12 diff.
Fold induction
miR relative expression
miR 133
miR 122
miR 221
pRILES
pRILES/133T
pRILES/122T
pRILES/221T
*

Figure S2

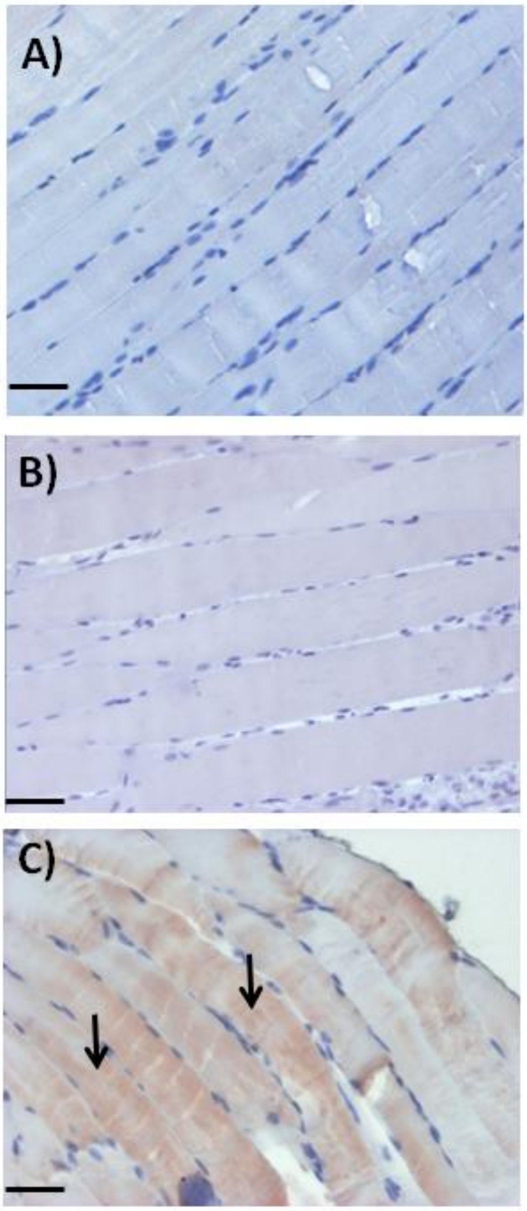

Figure S3

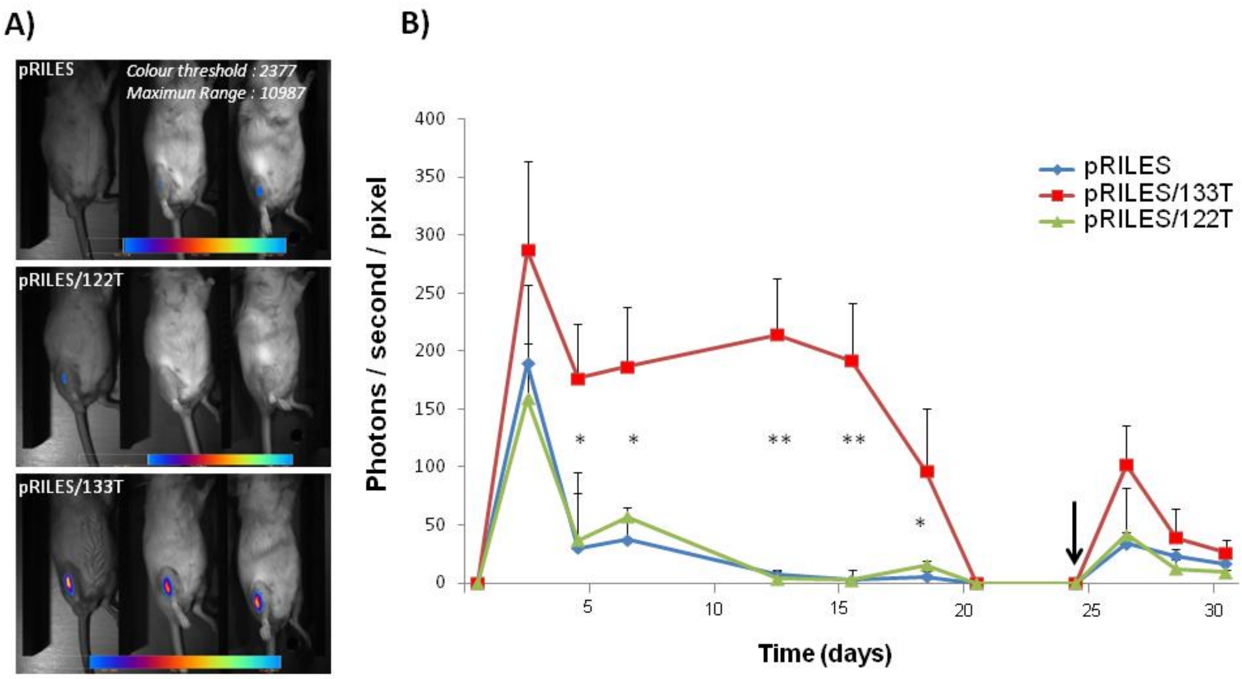
A)
pRILES
Colour threshold : 2377
Maximun Range : 10987
pRILES/122T
pRILES/133T
B)
Photons / second / pixel
Time (days)
pRILES
pRILES/133T
pRILES/122T
*
*
**
**
*
0
50
100
150
200
250
300
350
400
5
10
15
20
25
30

Figure S4

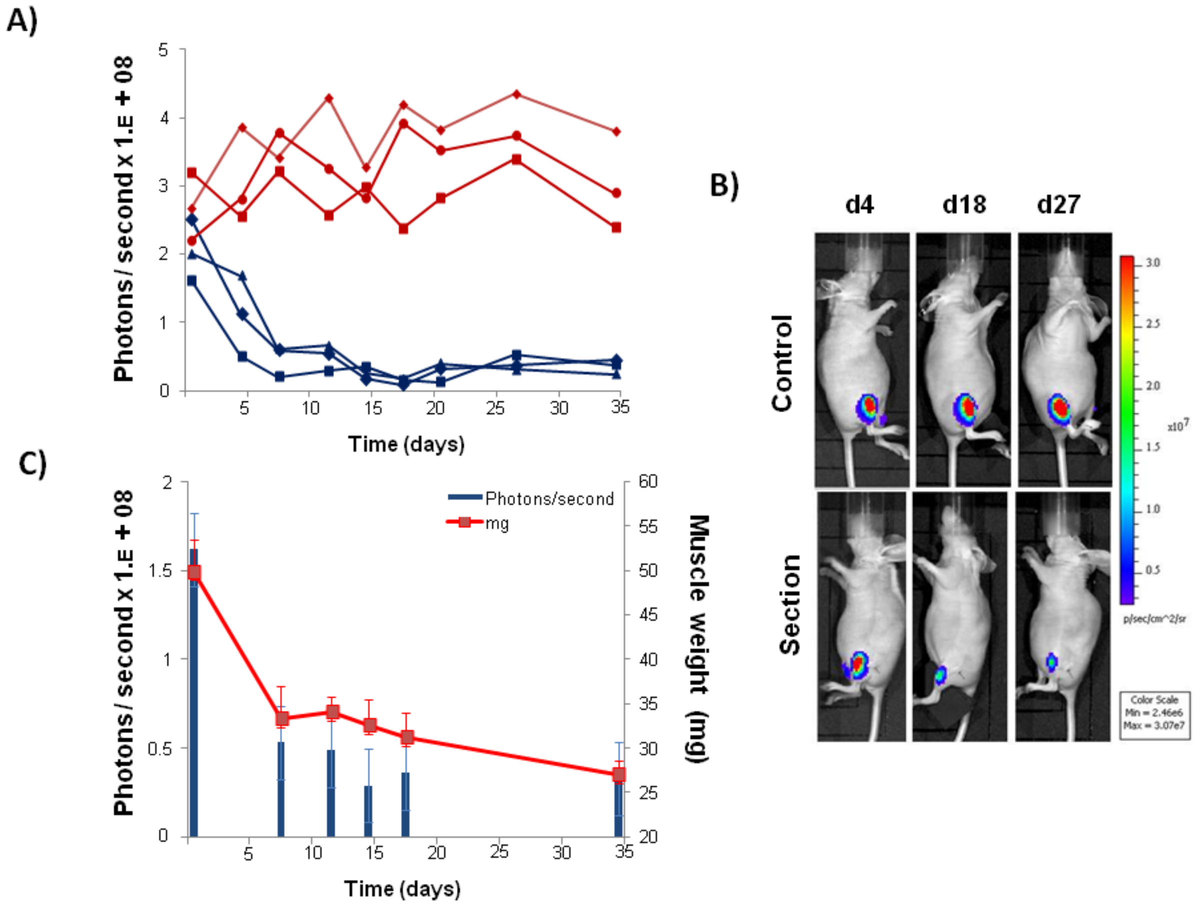
A)
Photons / second x 1.E + 08
0
1
2
3
4
5
5
10
15
20
25
30
35
Time (days)
B)
d4
d18
d27
Control
Section
3.0
2.5
2.0
1.5
1.0
0.5
x10^7
p/sec/cm^2/sr
Color Scale
Min = 2.46e6
Max = 3.07e7
C)
Photons/second
mg
Photons / second x 1.E + 08
0
0.5
1
1.5
2
Muscle weight (mg)
20
25
30
35
40
45
50
55
60
5
10
15
20
25
30
35
Time (days)

Figure S5

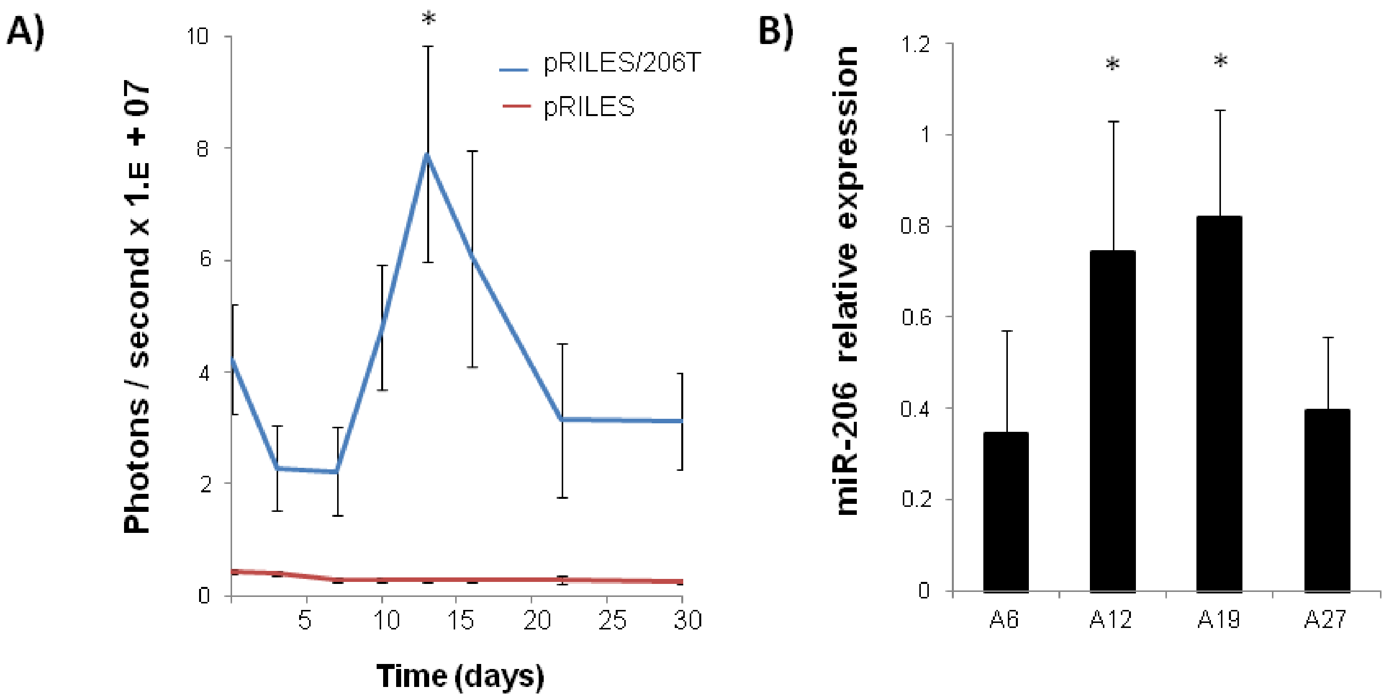
A)
10
8
6
4
2
0
5
10
15
20
25
30
*
pRILES/206T
pRILES
Photons / second x 1.E + 07
Time (days)
B)
1.2
1
0.8
0.6
0.4
0.2
0
*
*
miR-206 relative expression
A6
A12
A19
A27

Figure S6

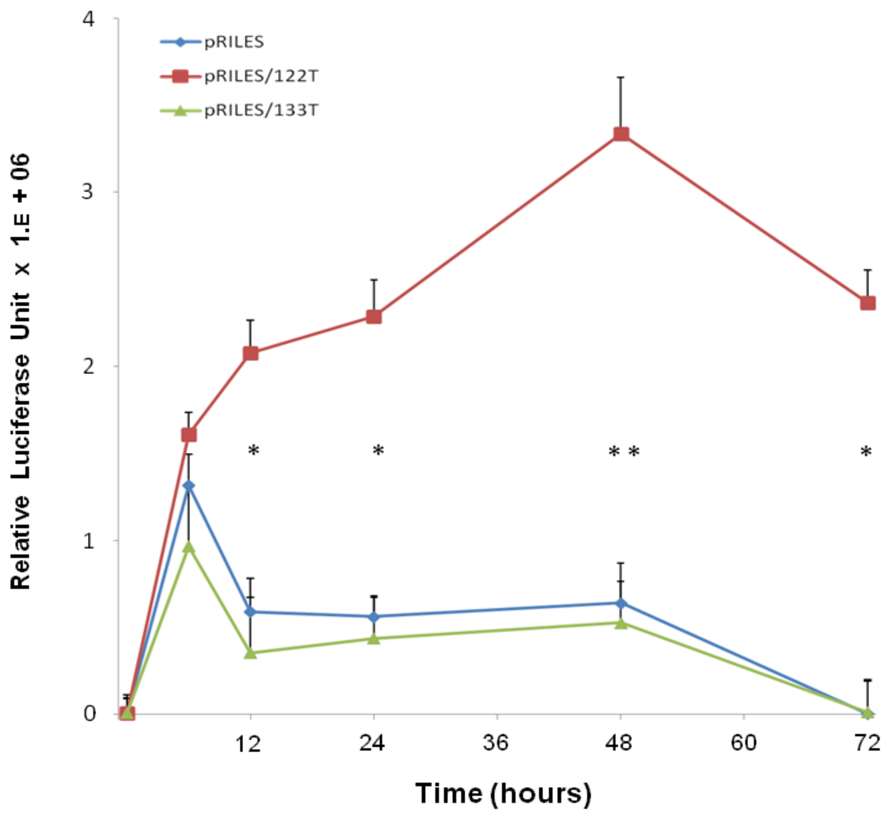
4
3
2
1
0
pRILES
pRILES/122T
pRILES/133T
Relative Luciferase Unit x 1.E + 06
*
*
* *
*
12
24
36
48
60
72
Time (hours)

Figure S7
